# Long non-coding RNAs in Oral squamous cell carcinoma: biologic function, mechanisms and clinical implications

**DOI:** 10.1186/s12943-019-1021-3

**Published:** 2019-05-27

**Authors:** Lei Zhang, Xiang Meng, Xin-wei Zhu, Deng-cheng Yang, Ran Chen, Yong Jiang, Tao Xu

**Affiliations:** 1 College & Hospital of Stomatology, Anhui Medical University, Key Lab. of Oral Diseases Research of Anhui Province, Hefei, 230032 China; 2 Department of Periodontology, College and Hospital of Stomatology, Anhui Medical University, Hefei, 230032 Anhui Province China; 3 School of Stomatology, Anhui Medical University, Hefei, 230032 Anhui Province China; 4 Outpatient Department of Binhu District, College and Hospital of Stomatology, Anhui Medical University, Hefei, 230601 Anhui Province China; 5 Department of Stomatology, The Fourth Affiliated Hospital of Anhui Medical University, 372 Tunxi Road, Hefei, 230000 Anhui Province China; 6 School of Pharmacy, Anhui Key Laboratory of Bioactivity of Natural Products, Anhui Medical University, 81 Meishan Road, Hefei, 230032 Anhui Province China; 7 Institute for Liver Diseases of Anhui Medical University, Anhui Medical University, 81 Meishan Road, Hefei, 230032 Anhui Province China

**Keywords:** Long non-coding RNAs, Oral squamous cell carcinoma (OSCC), Biomarker, Tumorigenesis

## Abstract

There is growing evidence that regions of the genome that cannot encode proteins play an important role in diseases. These regions are usually transcribed into long non-coding RNAs (lncRNAs). LncRNAs, little or no coding potential, are defined as capped transcripts longer than 200 nucleotides. New sequencing technologies have shown that a large number of aberrantly expressed lncRNAs are associated with multiple cancer types and indicated they have emerged as an important class of pervasive genes during the development and progression of cancer. However, the underlying mechanism in cancer is still unknown. Therefore, it is necessary to elucidate the lncRNA function. Notably, many lncRNAs dysregulation are associated with Oral squamous cell carcinoma (OSCC) and affect various aspects of cellular homeostasis, including proliferation, survival, migration or genomic stability. This review expounds the up- or down-regulation of lncRNAs in OSCC and the molecular mechanisms by which lncRNAs perform their function in the malignant cell. Finally, the potential of lncRNAs as non-invasive biomarkers for OSCC diagnosis are also described. LncRNAs hold promise as prospective novel therapeutic targets, but more research is needed to gain a better understanding of their biologic function.

## Introduction

Oral squamous cell carcinoma (OSCC), characterized by differentiation and a propensity for lymph node metastasis [1], is the sixth most common cancer worldwide with over 200,000 newly diagnosed once each year, and can be divided into three major subsites: buccal mucosal SCC (BMSCC), tongue SCC (TSCC), and lip SCC (LSCC) [2, 3]. Percentages of morbidity and mortality in males are 6.6/100,000 and 3.1/100,000 respectively, while in females, the same percentages are 2.9/100,000 and 1.4/100,000 [4]. Additionally, the incidence of OSCC is increasing among young white individuals age 18 to 44 years, particularly among white women [5]. Due to its risk factor exposure, low cure rate and high mortality, OSCC represents a global public health problem, with a great individual and socioeconomic burden.

The occurrence of OSCC is a complex multistep process. Normal oral keratinocytes are prolonged by adverse factors, resulting in intracellular microenvironment imbalance and genetic alterations. Genetically unstable precancerous keratinocytes can transfer these inheritable alterations to their clones [6]. These carcinogenic factors include key disorders on TP53, NOTCH1 (Notch homolog 1 genes are translocation-associated), EGFR (epidermal growth factor receptor), CDKN2A (cyclin-dependent kinase inhibitor 2a), STAT3 (signal transducer and activator of transcription 3), Cyclin D1 and retinoblastoma [7]. Normal oral keratinocytes are transformed into precancerous lesions through various signaling pathways, which further deteriorate into malignant tumors. Moreover, Yuan et al. [8] performed a case-control study including 444 OSCC cases and 984 healthy controls to investigate whether H19 genetic variants affect the risk of OSCC in the Chinese population. It was finally determined that the two SNPs, rs2839701 and rs217727, were related to OSCC susceptibility and indicated that the SNPs in H19 might be OSCC biomarkers.

Smoking and drinking are the two main causes of the high incidence of OSCC. A meta-analysis showed that smokers were at higher risk of developing oral cancers than non-smokers [9]. Cigarette smoke exerts inflammatory and suppressive effects on immune cells, alters mucosal immunity and promotes autoimmunity, resulting in oral cancers [10]. Yamashita et al. [11] found that smoking and drinking can inhibit 5-fluorouracil (5-FU)-related metabolic enzymes through the induction of dihydropyrimidine dehydrogenase (DPD; a sole catabolic enzyme of 5-FU) activity, which in turn lead to oral cancers. In addition, alcohol consumption can interact with the polymorphisms of ALDH2 and CYP2E1-RsaI genes to increase OSCC risk [12]. Human papillomavirus (HPV) is also considered as one of the potential risk factors of OSCC. As early as 2007, HPV 16 was recognized by the International Agency for Research on Cancer as a risk factor for OSCC. HPV33, HPV35 and others (also found in cervical cancer) have been also considered to trigger OSCC [13].

Despite advancements in diagnosis and treatment methods, 5-year survival rate has not improved significantly over the past decade, which ranges from 45 to 50% [14]. Surgical resection is considered to be a promising treatment strategy for early cancer [15]. However, recurrence after surgical resection is still a serious cause of cancer-related death [16]. Therefore, how to prevent postoperative recurrence and improve patient survival is still a major challenge in OSCC treatment. Increasing evidence shows that multiple oncogenes and tumor suppressor genes are involved in OSCC. This contributed to better understand the exact mechanisms between lncRNAs and OSCC, providing suitable approaches for clinical treatment. Meanwhile, the discovery of new cancer molecular targets can also effectively help to understand the pathogenesis and prognosis of OSCC.

**Fig. 1:**
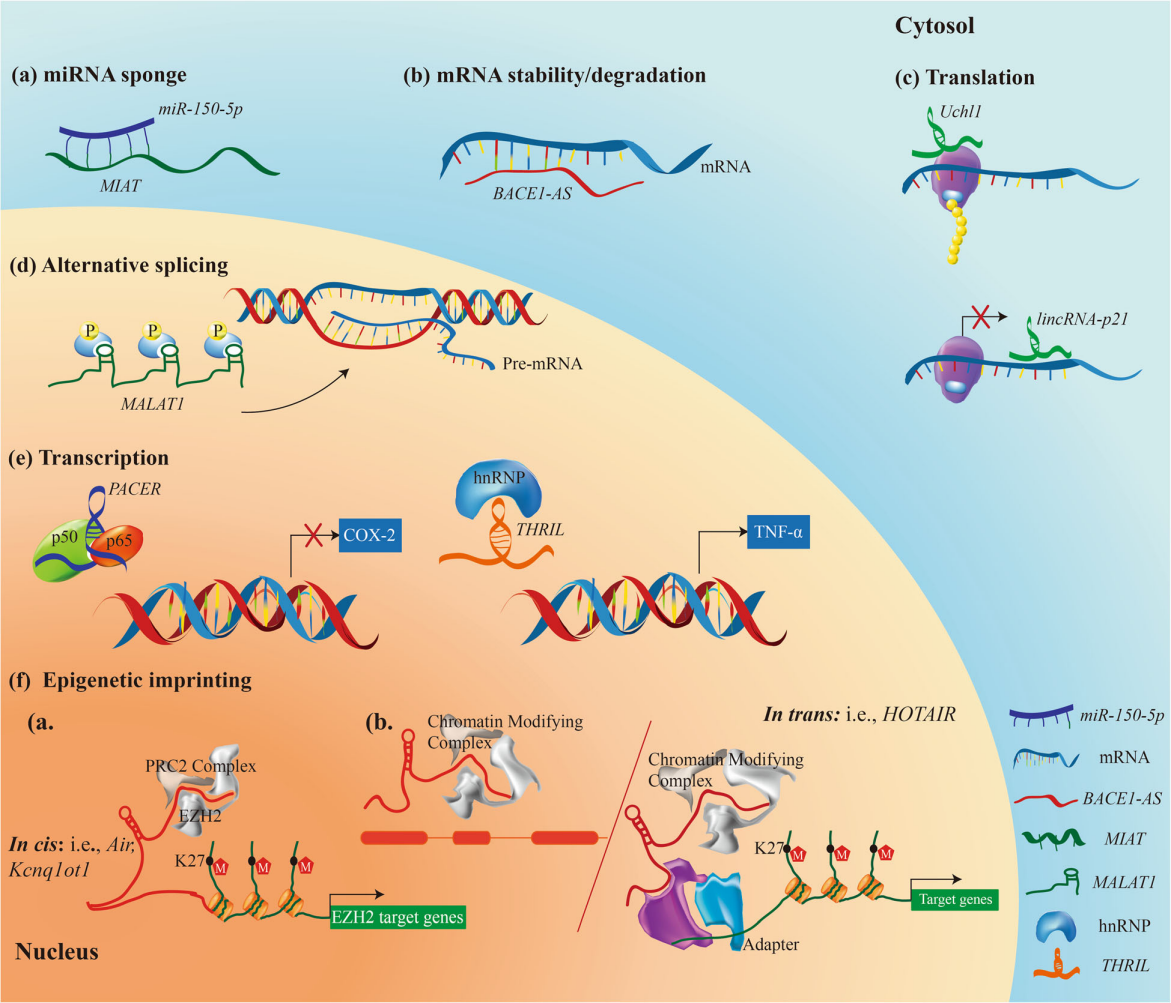
LncRNAs exert functions through a variety of signaling pathways in the human body. **a** miRNA sponge. MIAT, acting as a molecular sponge, binds to miR-150-5p, thereby upregulating the level of miR-150-5p target gene. **b** mRNA stability/degradation. LncRNA binding to mRNA may stabilize (e.g., BACE1-AS prevents miRNA-induced repression of BACE1 transcript) or decay target transcripts. **c** Translation. LncRNAs promote (like antisense Uchl) or repress (like lincRNA-p21) translation of transcripts. **d** Alternative splicing. MALAT1 acting as scaffold for SR proteins regulates pre-mRNA alternative splicing. **e** Transcription. PACER (lethe and p50-associated Cox-2 extragenic RNA) directly interacts with different subunits of NF-κB, thus preventing it from binding to the Cox-2 promoter. THRIL, together with heterogeneous nuclear ribonucleoproteins (hnRNPs), acts as RNA-protein complex and binds to TNF-α promoter and induces TNF-α expression. **f** Epigenetic imprinting. Working models of gene regulation by cis- (**a**) and trans-acting (**b**) lncRNAs. LncRNAs, such as Xist/RepA, Air, HOTAIR, and Kcnq1ot1, may act as docking platforms for the chromatin remodeling complex, polycomb repressive complex (PRC2) 2, which methylates histone H3 at lysine 27 (H3K27me3), leading to a repression or gain of transcriptional activity, respectively

Accumulating evidence showed that non-coding RNAs (ncRNAs), such as long non-coding RNAs (lncRNAs), played vital regulatory roles in the cellular physiological process [17–19] (Fig. 1). For example, lncRNAs could act as miRNAs sponge to weaken regulations of miRNAs on mRNAs [20, 21]. Regulatory mechanism of lncRNA-induced oral cancers, especially OSCC, should not be ignored. LncRNAs associated with cancer pathogenesis were primarily involved in cellular macromolecules (including chromatin, protein, RNA) [22, 23]. So far, no perfect diagnostic marker of OSCC has appeared. Aberrant expression of some lncRNAs had been shown to be closely correlated with cancer prognosis. In OSCC tissues, HOTAIR (HOX transcription antisense RNA) was highly expressed, and its expression level was correlated with tumor size, TNM (Tumor Node Metastasis) stage, and prognosis of OSCC [24]. This suggested that HOTAIR could be employed as a biomarker for diagnosis and prognostic determination as well as a molecular target for therapy. On the one hand, epigenetic changes in a body (mainly involved in DNA methylation, histone modifications and modifications in micro ribonucleic acids) can provide valuable biomarkers [25]. On the other hand, comparing seven mRNAs and three proteins in saliva, OSCC patients had a higher level of interleukin (IL)-8 and subcutaneous adipose tissue than healthy controls [26]. This result suggested new biomarkers. Moreover, Tang et al. [27] found that MALAT-1 and HOTAIR in saliva samples from OSCC patients could be expressed in patients with primary tumor. It was worth noting that the expression level of HOTAIR in saliva of OSCC metastatic patient was different from that of primary tumor controls. This suggested that the detection of lncRNAs in saliva can be used for clinical non-invasive and rapid diagnosis of OSCC, and to determine whether there was metastasis. At present, most of the clinical treatment programs (chemoradiotherapy, surgery, EGFR inhibitors and COX-2 inhibitors, and photodynamic therapy) are high economic cost and highly damaging treatment, which are a burden for patients and society [28, 29]. Nanotechnology-Based approaches for prevention and therapy have become a hot spot and need further clinical validation [30]. In general, an effective, safe and prognostic treatment is urgently needed for the therapy of OSCC. Therefore, precise treatment is a must. The role of ncRNAs, particularly lncRNAs, in cancer is gradually being amplified, and the underlying mechanism between them and OSCC needs to be clarified [31]. Therefore, this review aims to elucidate the mechanism of lncRNAs regulation in OSCC patients, and to explore a new approach for better clinical treatment of diseases.

## Overview of lncRNA

LncRNAs are a novel class of ncRNAs and pervasively transcribed in the human genome. Most (but not all) lncRNAs are synthesized by RNA polymerase II and share many of the biological characteristics of mRNAs, though they bear little or no coding potential [32]. Unlike siRNAs and miRNAs whose sizes are usually comprised between 20 to 24 nucleotides, lncRNAs range in size from 200 to more than one hundred thousand nucleotides and are always capped and polyadenylated [33, 34].

**Fig. 2:**
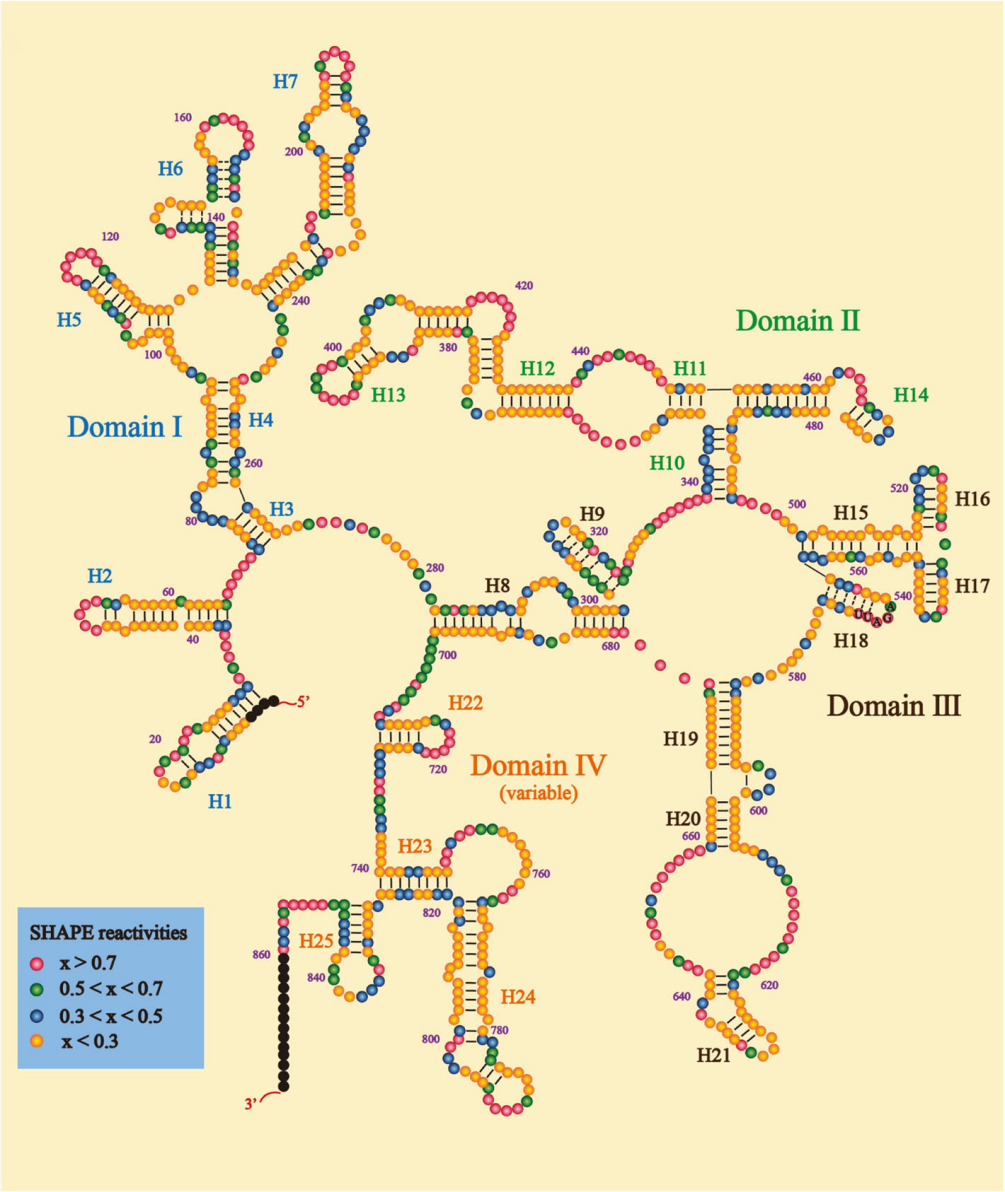
The steroid receptor RNA activator (SRA) lncRNA contains 4 subdomains. The human SRA has a length of 870 nt, organized into 4 sub-domains and 25 helices. Four biochemical techniques (SHAPE, in-line, DMS and RNase V1) were used to obtain the secondary structure. Blue, domain I; green, domain II; black, domain III; yellow, domain IV

The large number of lncRNAs, large molecular weight and poor stability in vitro have hindered the revealing of its structure. The nucleotide sequence of lncRNAs constitutes its primary structure. LncRNAs can regulate transcriptional translation of a target gene directly or a gene upstream or downstream of a target gene indirectly by binding to a target gene by base-complementary pairing [35], like lncRNA Gas5 [36] and lncRNA 1/2-sbsRNAs [37]. Meanwhile, a major feature of lncRNAs is a propensity to fold into thermodynamically stable secondary and higher-order structures. Hydrogen bonds formed by internal structure of RNA (including the Watson-Crick face, the Hoogsteen and ribose face) together construct the secondary its structure that include double helices, hairpin loops, bulges and pseudoknots [38, 39] (Fig. 2). The secondary and higher-order structures of RNA appear to play their primary biological function. For example, p53 is activate by motifs M2 and M3 that are secondary folding motifs of lncRNA MEG3 (maternally expressed gene 3) isoforms, rather than its primary sequence [40].

By using antisense oligonucleotides and RNA interference, it has been shown that lncRANs are present in different parts of the cell, including the nucleus (such as: MALAT1 and NEAT1), cytoplasm (such as: DANCR and OIP5-AS1) or both (such as: TUG1, CasC7 and HOTAIR) [41]. Technological advances have enabled us to explore the vital roles of the lncRNA family (Table 1) [42–63]. Specifically, they were mainly divided into in vivo (DMS-seq, Structural-seq, and Mod-seq, icSHAPE, CLASH and hiCLIP) and in vitro (SHAPE-seq, SHAPE-MAP, and RING-Map, PARS and FragSeq, ss/dsRNA-seq Techniques) methods [64]. These techniques, especially combined with high-throughput sequencing, were also speculated to investigate lncRNA multi-level structures [65, 66].

**Table 1:** Main methods to detect and quantify noncoding RNAs

	Method	Advantages	Limitations	Reference (PMID)
ncRNA	Northern blot	Gold standard; Specificity	Limited sensitivity; Low throughput; Time consuming; Limited for quantification	([42], 11679671); ([43], 18025253)
ncRNA	SPR	Sensitivity	Expensive read-out system; High background signal; Limited throughput	([44], 17061884); ([45], 21284927)
ncRNA	In situ hybridization	Locates miRNA in tissue and cell compartments	Low throughput; Invasive sample collection; Limited sensitivity; Limited quantification	([46], 16369549); ([47], 22482439)
ncRNA	Microarray	High throughput	Fair specificity; Medium sensitivity; Limited quantification	([48], 17675362); ([49], 22593088)
ncRNA	Bioluminescence	Sensitivity	High costs; Difficult to standardize	([50], 18302417)
ncRNA	Electrochemical detection	Cost-effective sensitive sensors	Verified background signal; Special nanoparticle labels	([51], 19367400); ([52], 21207998)
ncRNA	RNA sequencing	High throughput; Sensitivity; Specificity	Complex data analysis; High costs	([53], 20473869); ([54], 22298638)
ncRNA	Nanopore-based RNA detection	Single-molecule detection; Contractible; Possibilities for high throughput; Rapid	Requiring sophisticated detection instruments; Complex data analysis	([55], 20972437); ([56], 21892163)
ncRNA	qRT-PCR	Semi-high throughput; Good quantification; Amplification enables; Sensitivity	Difficult to distinguish single-nucleotide differences; Not for ncRNA discovery	([57], 21867561); ([58], 22332658)
ncRNA	Flow cytometry-FISH	High throughput detection of in situ hybridization	No quantification; No location of the ncRNA	([59], 22057868)
ncRNA	Nanoresonator chip	Quantitative sensitivity; Specificity	Limited reproducibility; Complex production process for nanoresonators	([60], 22115599)
ncRNA	Base stacking hybridization coupling with time-resolved fluorescence technology	Rapid, Universal label; Sensitivity	Needs fluorescent tag	([61], 22365748)
ncRNA	Scanometric miRNA array	Sensitivity	High background signal	([62], 22489825)
ncRNA	Fluorescence quenching on graphene oxide	Amplification process; Sensitivity	High costs	([63], 22510208)

*LNA* Locked nucleic acid, *ncRNA* Noncoding RNA, *qRT-PCR* Quantitative reverse transcription PCR, *LOD* Limit of detection, SPR Surface plasmon resonance

Accompanying with the increasing number of lncRNAs, they can be mainly divided into the following categories according to different characteristics: (1) genome location and context (intergenic lncRNAs and intronic lncRNAs, sense and antisense lncRNAs), (2) exerted an effect on DNA sequences (cis-lncRNAs, trans-lncRNAs), (3) mechanism of functioning (transcriptional regulation, post-transcriptional regulation and other mechanisms of lncRNA functioning), (4) targeting mechanism [67, 68].

To date, it has demonstrated lncRNAs could regulate multiple disease progressions. For instance, 4313 lncRNAs were upregulated and 4612 lncRNAs were downregulated in periodontitis by using RT-PCR [69]. Additionally, the upregulation of lncRNA SNHG20 and the downregulation of DLEU1 (deleted in lymphocytic leukemia 1) were stably correlated with the progression of OSCC. Nishiyama et al. [70] found DLEU1 silencing suppressed migration, invasion, and xenograft formation in OSCC cells, which was suggestive of its oncogenic functionality. Another lncRNA, UCA1 (urothelial cancer associated 1), also was upregulated in OSCC and enhanced proliferation and metastasis of OSCC cells [71], which was similar to consequences of other cancers in lung [72], stomach [73] and bladder [74].

## LncRNA-centric targeting regulation

**Fig. 3:**
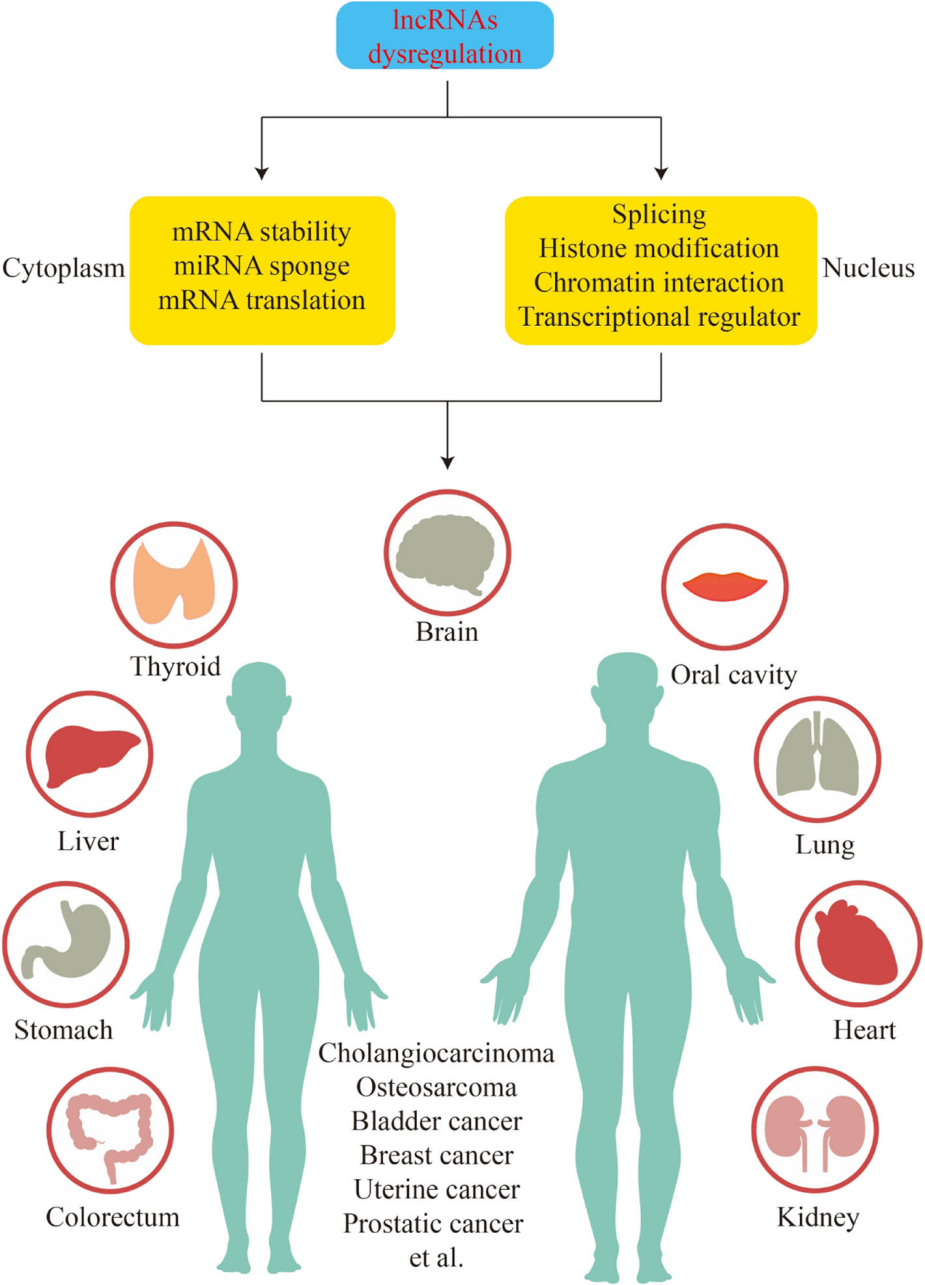
LncRNAs dysregulation is associated with a variety of diseases in humans. LncRNAs can affect human health through a variety of signaling pathways which can be divided into cytoplasmic signaling pathways and intranuclear signaling pathways. Cytoplasmic signaling pathways include mRNA stability, miRNA sponge and mRNA translation. Intranuclear signaling pathways include splicing, histone modification, chromatin interaction and transcriptional regulator

LncRNAs can mediate chromatin remodeling and transcription regulation, mainly as signals, decoys, guides, and scaffolds, resulting in the downregulation or upregulation of target genes and triggering various diseases [75, 76] (Fig. 3). For example, lncRNA can interact with DNA. The locus 515 kb upstream of MYC can transcribe CCAT1-L, which can interact with MYC transcriptional regulation and accelerate long-range chromatin looping. Specifically, *in cis* overexpression of CCAT1-L triggers tumorigenesis through promoting MYC expression [77]. In addition, lncRNA-RNA interactions and lncRNA-protein interactions together constitute a complex regulatory network of lncRNA to control occurrence and development of cancers [78, 79]. Xu et al. [80] elaborated that various lncRNAs are involved in the pathogenesis of prostate cancer and can be used as biomarkers for diagnosis, treatment and prognosis. This also suggested that lncRNAs can play a similar role in OSCC.

**Fig. 4:**
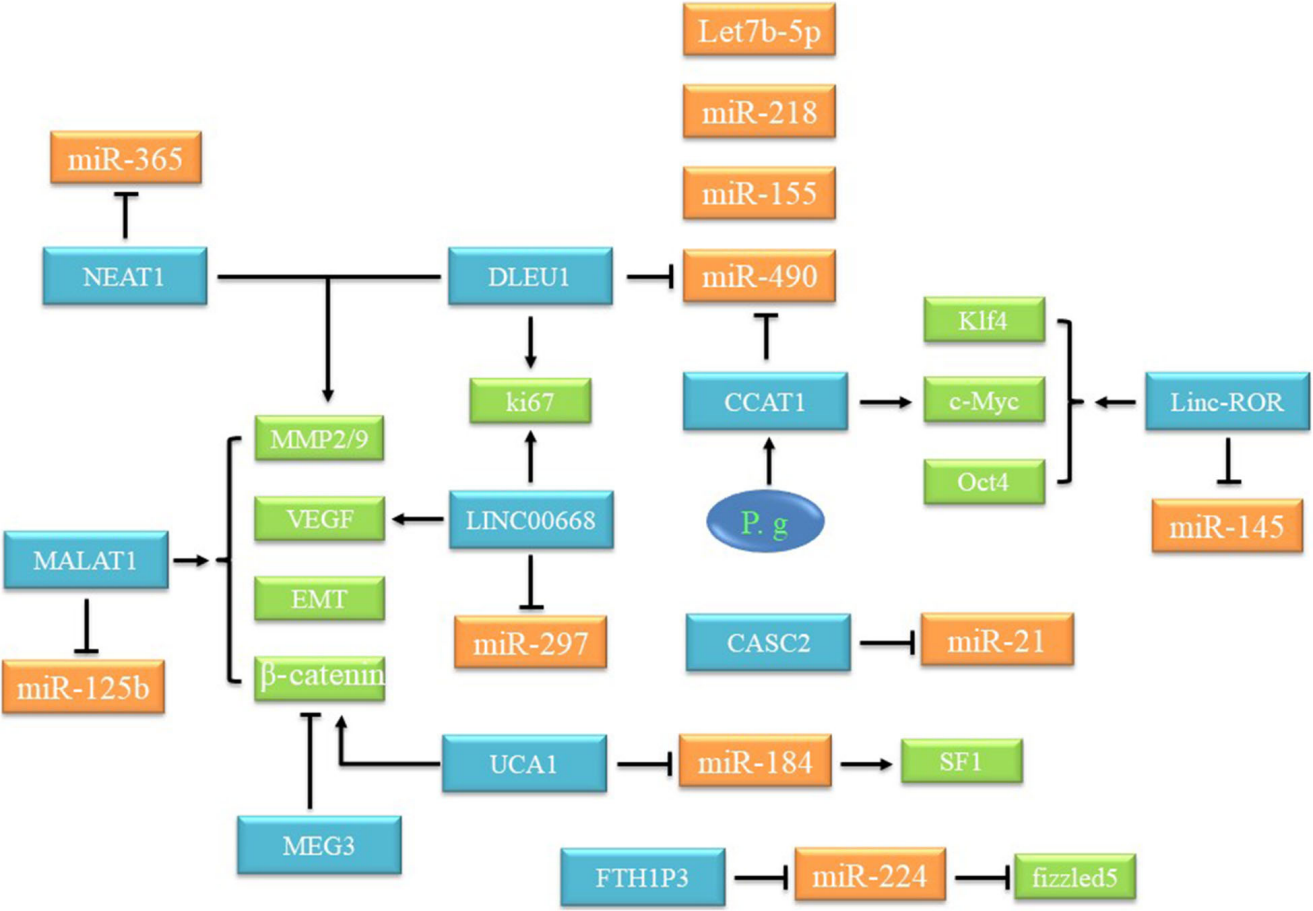
Overview of the role of lncRNAs with miRNAs in OSCC cells

“Competing endogenous RNA (ceRNA)” have been proposed to emphasize regulatory dialogues between different RNAs, including lncRNAs, miRNAs, transcribed pseudogenes, and circular RNAs (circRNAs) [81]. Particularly, the focus of interactions between miRNAs and lncRNAs in various human disease progression is gaining attention. MiRNAs, composed of 19–25 base pairs, mainly target protein-coding genes at the post-transcriptional level [82]. For example, Hsa-miR-1 suppresses expression of the UCA1 via an Ago2-slicer-dependent signaling and structure recognition 3-untranslated regions (3-UTRs) of UCA1 to play tumor suppressive roles [83]. Similar and different mechanisms also occur in pancreatic cancer [84], breast cancer [85] and colorectal cancer [86]. Generally speaking, lncRNAs exert “sponge-like” effects on various miRNAs to inhibit miRNA-mediated functions (Fig. 4). However, regulatory networks of lncRNAs still have unknown areas.

## Biologic functions of lncRNAs in OSCC

The carcinogenesis of OSCC is a multifactorial and multistep process, involving various aspects such as genes, epigenetics and environment. With the continuous exploration and clarification of the structure and function of lncRNAs, the dysregulation of lncRNAs has become a non-negligible regulatory element for the development of cancer [87]. Hereby, this review will elaborate on the regulation of lncRNAs in OSCC (Table 2) [70, 88–106].

**Table 2:** The expression of LncRNA in OSCC

LncRNA	Cytology	Location	Expression	Function in tumorigenesis	Reference (PMID)
MALAT1	11q13.1	Tca8113, SCC-25, CAL-27 and HN5 cells	+	Biomarker	([88], 26522444); ([89], 28926115)
CCAT1	8q24.21	OSCC tissues/HIOECs	+	Biomarker	([90], 28413645); ([91], 28286742)
MEG3	14q32.3	OSCC tissues/SCC-15 and CAL-27 cells	–	Biomarker, Tumor suppressor	([92], 25351956); ([93], 28959364)
UCA1	19p13.12	SCC-15 and CAL-27/Tca8113, TSCCA, CAL-27 and SCC-9 cells	+	Biomarker, Oncogene	([94], 27560546); ([95], 29125238)
AC132217.4	–	UM-SCC6H and SCC-090 cells	+	Biomarker	([96], 28823965)
HNF1A-AS1	12q24.31	OSCC tissues and cell lines	+	Oncogene	([97], 30404566)
HAS2-AS1	8q24.13	SCC-9 and CAL-27 cells	+	Biomarker	([98], 28485478)
HOTAIR	12q13.13	TSCCA, Tca8223, KB and CAL-27 cells	+	Biomarker, Oncogene	([99], 25901533); ([100], 30053324)
Linc-RoR	18q21.31	OSCC tissues	+	Biomarker	([101], 28443494)
LINC00668	18p11.31	SCC-4, SCC-9, SCC-1, SCC-25, TU-183, HSU-3, FADU, OEC-M1, SNU-1041, SCC-15 cells	+	Oncogene	([102], 28564590)
NEAT1	11q13.1	HN-4, Tca-8113, UM-SCC-1, CAL-27, SCC-25 and SCCKN cells	+	Biomarker	([103], 30186464)
FTH1P3	2p23.3	SCC-4, SCC-9, SCC-1, SCC-25, TU-183, HSU-3, FADU, OEC-M1, SNU-1041, and SCC-15 cells	+	Oncogene	([104], 28093311)
DLEU1	13q14.2-q14.3	SAS, Ca9–22, HSC-3, KON, MOT, HSC-4, OSC-19 and MON2 cells	+	Biomarker, Oncogene	([70], 30069008)
CASC2	10q26.11	SCC-090 and SCC-25 cells	–	Tumor suppressor	([105], 30467776)
FLJ22447	14q23.1-q23.2	HSC-3 cells	+	–	([106], 29346528)

Upregulation: +; Downregulation: -

## Molecular mechanisms of lncRNAs in OSCC

MALAT1, mapped to human chromosome 11q13 and 8.7 kb long, was a highly conserved lncRNA and was also referred to as NEAT2 [107–109]. The triple helix-structure at the 3’end of MALAT1 given it unique stability [110, 111]. MALAT1 might interact with SR (serine/arginine-rich) splicing factors (including SRSF1, 2, and 3), which were involved in exon recognition and alternative splicing, to regulate alternative splicing of a subset of pre-mRNAs [112, 113]. MALAT1 was also involved in transcriptional and post-transcriptional regulation [114]. Moreover, binding of methylated and unmethylated Polycomb 2 protein to MALAT1 controlled relocation of growth control genes between Polycomb bodies and interchromatin granules [115]. It had been observed that level of MALAT1 was aberrant in some human tumors [107, 114, 116], and its expression level was associated with tumor recurrence and metastasis. Studies had shown that MALAT1 promoted cellular proliferation by regulating the activity of the E2F1 transcription factor, and then enhanced tumorigenesis [117, 118]. E2F1 transcription factor affected cell cycle regulation and apoptosis [119]. Meanwhile, depletion of MALAT1 affected activity of the oncogenic transcription factor B-MYB (MYBL2), blocking cell cycle in G1/S phase, thereby reducing cell proliferation [118, 120]. B-MYB was a physiological regulator of cell cycle progression, cell survival and cell differentiation, and its overexpression was associated with poor patient outcome in numerous cancers [121]. These studies indicated that MALAT1 played important functions in a string of biological processes.

Emerging evidence suggested that epithelial–mesenchymal transition (EMT), an indispensable mechanism during morphogenesis, was also a crucial event in OSCC. After SDF-1/CXCR4 system induction, EMT may activate PI3K-AKT/PKB signaling pathway to participate in the lymph node metastasis of OSCC [122]. When MALAT1 was deleted in OSCC cell lines TSCCA and Tca8113, EMT mediated cell migration and invasion were inhibited. The low level of MALAT1 weakened β-catenin and NF-κB signaling pathways in OSCC. Correspondingly, tumor growth in the Tscca xenograft model was also inhibited [88]. In addition to MALAT1 increased, STAT3 was also overexpressed, while miR-125b was downregulated in OSCC cell lines [123]. STAT3 was a molecule in the OSCC inflammation-mediated/related carcinogenesis signaling pathways. It can regulate the expression of various genes to cope with cellular stimuli. Meanwhile, STAT3 and NF-κB interacted with each other to regulate cell tumor angiogenesis and invasiveness [124]. Furthermore, miR-125b can directly bind to the 3′-UTR of STAT3, and then decreased the protein levels of STAT3 in MG-63 and Saos-2 cells, suggesting that STAT3, as the functional downstream target of miR-125b, played a role in the transcriptional activation of miR-125b [125]. MALAT1 attenuated the tumor suppressive effect of miR-125b mimics by up-regulating STAT3. The established nude mouse model was further confirmed that upregulated MALAT1 played an oncogene role in OSCC via miR-125b/STAT3 axis [89]. These newly discoveries need further investigation to exploit mechanistic insights of MALAT1 in OSCC progress.

Colon Cancer Associated Transcript 1 (CCAT1), also known as a cancer-associated region lncRNA-5 (CARLo-5) or CCAT1-S with a length of 2628 nucleotides, located in chromosome 8q24.21 [77]. CCAT1 contained two exons and a poly-A tail and was mainly expressed in the nucleus. On the one hand, the CCAT1-L locus is located within a strong super-enhancer that consist of large clusters of transcriptional enhancers formed by binding of master transcription factors/mediators and to be associated with genes that control and define cell identity [77]. On the other hand, CCAT1 closed to c-Myc, a well-known oncogenic transcription factor, and was triggered by c-Myc, resulting in cell proliferation and invasion enhancing [126]. C-Myc, one of the Myc proto-oncogene family members, was found a positive correlation with STAT3 [127]. CCAT1 could trigger c-Myc overexpression through its ceRNA activity on miR-155 [128]. It was found that the high level of CCAT1 downregulated miR155-5p, let7b-5p, miR490-3p by a sponging mechanism and miR218-5p by epigenetic silencing [21, 129]. CCAT1 had been shown to be overexpressed in a variety of cancers and a rising star of oncogenic lncRNAs [128, 130, 131].

Arunkumar et al. [90] collected 60 OSCC tumor samples and eight normal tissue samples, and found that c-Myc was also overexpressed in CCAT1 overexpressing tumor tissues, while miR155-5p and let7b-5p were downregulated. MiR218-5p and miR490-3p were also low expressed due to CCAT1 acted as a sponge. Moreover, miR-155-5p inhibitor, as an EMT suppressor, suppressed the STAT3 signaling pathway and increased suppressor of cytokine signaling 1 (SOCS1) expression. Suppressor of cytokine signaling 1 (SOCS1) recently served as a novel miR-155 target in breast cancer, and might also exerted roles in OSCC [132]. Geng et al. [91] explored the potential effects of *Porphyromonas gingivalis* (*P. gingivalis*) on OSCC and found that long-term stimulation of *P. gingivalis* promoted cell proliferation, accelerated cell cycle and promoted cell migration and invasion abilities. Further, CCAT1 was upregulated by using validation of microarray and proteomic assay. Long-term exposure of *P. gingivalis* can trigger tumor-associated molecules, such as CCAT1, to enhance tumorigenic properties of human immortalized oral epithelial cells (HIOECs) and participate in the pathogenesis of OSCC [91].

MEG3, the first to be found to have tumor suppressive effects, was a maternally imprinted gene located on chromosome 14q32.3 within DLK1–MEG3 locus [133, 134]. It was also called gene trap locus2 (Gtl2) located at chromosome in mouse [135]. The MEG3 gene, was controlled by two differentially methylated regions (DMRs) that comprised of multiple methylated CpG sites: the intergenic DMR (IG-DMR) and the MEG3-DMR [136]. Multiple signaling pathways were involved in MEG3 inhibition of cell proliferation and metastasis [137–139]. Elevated levels of MEG3 or/and miRNA-26a inhibited cell proliferation, suppressed cell cycle progression and induced cell apoptosis [140].

After treating HOK cells with arecoline, Shiah et al. [92] found a significant decrease in MEG3 and 14q32.2 miRNAs. While Wnt-7b overexpressed, the phosphorylation of GSK-3β and active-β-catenin in DOK cells were markedly enhanced, causing cyclin D and c-Myc upregulated [92]. Cyclin D_1_ was an indispensable nuclear protein in the G_1_/S phase of the cell cycle. Bova et al. founded that cyclin D_1_ overexpression and cyclin D_1_ gene amplification in OSCC [141]. In OSCC cells, multiple signaling pathways, including DNA methylation, downregulated MEG3 expression. MEG3 decreased in OSCC cells by using RT-qPCR technique. Low expression of MEG3 significantly increased Cal27 cell proliferation when compared with control group, suggesting that MEG3 suppressed OSCC cell proliferation. Meanwhile, OSCC cell apoptosis was inhibited, and metastasis was promoted [93]. Wnt/β-catenin signaling pathway, one of the classical signaling pathways in the process of cell signal transduction, was involved in cancer cell proliferation, migration, invasion, tumorigenesis and metastasis [142, 143]. Taken together, MEG3 was found that it could inhibit the Wnt/β-catenin axis to act as a tumor suppressor [93].

Urothelial carcinoma-associated 1 (UCA1) was located on human chromosome 19p13.12 and contained three exons and two introns. UCA1 had three isoforms, including 1.4 kb, 2.2 kb, and 2.7 kb in length, generated by splicing and polyadenylated [144]. The 1.4 kb isoform was labeled as lncRNA UCA1; the 2.2 kb isoform was labeled lncRNA UCA1a or lncRNA CUDR; while the biological role of the 2.7 kb isoform was not known [145]. Additionally, UCA1 may affect CREB expression and activity through PI3K-AKT dependent pathway, and then regulated cell cycle progression [146]. The PI3K/AKT/mTOR signaling pathway was activated in various cancers via stimulation of proliferation, survival, metabolic reprogramming, and invasion/metastasis, as well as suppression of autophagy and senescence [147, 148]. Ectopic expression of lncRNA UCA1 in bladder cancer cell line BLS-211 demonstrated that UCA1 was oncogenic [149]. Studies had found UCA1 was dysregulated and participated in the development of a few cancers including hepatocellular carcinoma [150], pancreatic cancer [145], bladder cancer [74].

Yang et al. [94] concluded that UCA1 was upregulated from 140 TSCC tissue samples. The results demonstrated that tumor growth was inhibited in vivo after UCA1 was deleted. In addition, UCA1 silencing inhibited cell proliferation, migration and invasion in OSCC cell lines. Correspondingly, UCA1-si could suppress OSCC cell proliferation in vitro via the CCK-8 assay. Further analysis found UCA1 upregulation could activate the Wnt/β-catenin signaling pathway [94]. In another study, the results suggested UCA1 overexpressed in OSCC tissues, cell lines, and Cisplatin (CDDP)-resistant OSCC cells by qRT-PCR [95]. CDDP was an anti-tumor drug that was clinically used to treat OSCC [151]. After UCA1 was knocked by UCA1-siRNA, CDDP chemoresistance weakened, suggesting that UCA1 facilitated proliferation, restrained apoptosis and conferred CDDP resistance of OSCC cells. Luciferase reporter assay showed UCA1, as a ceRNA, downregulated its expression and upregulated steroidogenic factor-1 (SF-1), an essential regulator of tissue-specific gene expression in steroidogenic cells, via sponging miR-184 in OSCC cells [95]. However, the inter-regulation between UCA1 and miR-184 needed intermediate-the store-operated Ca^2+^ entry (SOCE), but its role between the two were unknown [152].

Interestingly, AC132217.4 was another upregulated lncRNA in OSCC samples. Additionally, krüppel-like factor 8 (KLF8) and insulin-like growth factor 2 (IGF2) have also been overexpressed. KLF8, one of the krüppel-like C2H2 zinc-finger transcription factor family proteins, was considered to exert roles in cancer initiation and progression [153, 154]. IGF2 was an anti-apoptotic endocrine protein, and its upregulation existed in many cancers [155]. Elevated serum IGF2 was proven to be associated with increased risk of developing various cancers including colorectal, prostate and lung [156]. But the regulatory mechanisms between KLF8 and IGF2 were still unknown. AC132217.4 could upregulate IGF2 levels by interacting with 3’UTR of IGF2 mRNA. In addition, cell migration and EMT are promoted [96]. It found that transcription factor STAT3 could positively regulate HNF1A-AS1 levels, and Notch1 and Hes1 (the core factors of Notch signaling pathway) interacting with STAT3 could upregulate HNF1A-AS1 to accelerate OSCC progression [97]. HAS2-AS1 could stabilize HAS2 to promote hypoxia-induced EMT of OSCC cells [98].

HOTAIR regulated E-cadherin through binding oncogene enhancer of zeste homolog 2 and H3K27me3, and it had a negative association with E-cadherin [99]. E-cadherin, expressed in most epithelial cells, was a calcium-dependent transmembrane glycoprotein, and it decreased in patients with OSCC [157, 158]. HOTAIR deletion resulted in downregulating expression of MAP 1LC3B (microtubule-associated protein 1 light chain 3B), beclin1 and autophagy-related gene (ATG) 3, and then autophagy was inhibited. The proliferation and metastasis ability of OSCC cells was also correspondingly weakened [54]. Regarding autophagy, these three molecules each play a complementary function. MAP 1LC3B, an essential protein for autophagosome elongation, were elevated in the tumor tissues of three subsites [159]. Beclin-1 may regulate autophagy process by forming the beclin-1 interactome with some co-factors such as Class III phosphatidylinositol 3-kinase (PI3KCIII)/Vps34, Vps15 [159]. ATG 3 belonged to autophagy-related proteins (ATGs) that regulated the autophagy process in the body [160].

Linc-RoR was first reported to be overexpressed in OSCC tumor specimens [101]. Meanwhile, downregulation of miR-145-5p and overexpression of c-Myc, Klf4, Oct4 and Sox2 indicated the existence of linc-RoR-mediated regulatory network [101]. MiR-145–5p, downregulated in several tumors, was a one of the crucial tumor suppressors, and be proposed as an important regulator of Sox2 [161]. It further suggested that linc-RoR and CCAT1 may share a partially coincident signaling pathway. OSCC tumorigenesis was deteriorated by overexpressed LINC00668 via miR-297/VEGFA axis. However, the mechanism was still to be further clarified [102]. Zhang et al. [104] found that lncRNA ferritin heavy chain 1 pseudogene 3 (FTH1P3) could serve as a molecular sponge of miR-224-5p to modulate fizzled 5 expression, an oncogene in OSCC cells, and facilitate OSCC progression. LINC00152 was found to be at a higher level in OSCC patient tissues. To further explore the role of LINC00152 in OSCC cells, Li et al. [162] transfected SCC9 cells with the sh-LINC00152 plasmid to decrease LINC00152 levels, and LINC00152 knockdown inhibited the proliferation of SCC-9 cells in turn. Moreover, cell proliferation, colony formation, migration, invasion, and the epithelial to mesenchymal transition were inhibited in vitro, as well as tumor growth was disturbed in vivo. A negative correlation between LINC00152 and miR-139 levels indicated LINC00152 acted as a miRNA sponge for miR-139-5p in OSCC [162]. Their targets are stated in the Table 3 [70, 88–96, 99–105].

**Table 3:** The targets of LncRNAs in OSCC

LncRNA	Targets	Location	Reference (PMID)
MALAT1	miR-125b	Tca8113, SCC-25, CAL-27 and HN5 cells	([88], 26522444); ([89], 28926115)
CCAT1	miR155-5p, let7b-5p, miR490-3p, miR218-5p	OSCC tissues/HIOECs	([90], 28413645); ([91], 28286742)
MEG3	miR-26a	OSCC tissues/SCC-15 and CAL-27 cells	([92], 253519560; ([93], 28959364)
UCA1	miR-184	SCC-15 and CAL-27/Tca8113, TSCCA, CAL-27 and SCC-9 cells	([94], 27560546); ([95], 29125238)
AC132217.4	IGF2	UM-SCC6H and SCC-090 cells	([96], 28823965)
HOTAIR	EZH2 and H3K27me3, MCL-1	TSCCA, Tca8223, KB and CAL-27 cells	([99], 25901533); ([100], 30053324)
Linc-RoR	miR-145-5p	OSCC tissues	([101], 28443494)
LINC00668	miR-297	SCC-4, SCC-9, SCC-1, SCC-25, TU-183, HSU-3, FADU, OEC-M1, SNU-1041 and SCC-15 cells	([102], 28564590)
NEAT1	miR-365	HN4, Tca8113, UM-SCC-1, Cal-27, SCC-25 and SCCKN cells	([103], 30186464)
FTH1P3	miR-224-5p	SCC-4, SCC-9, SCC-1, SCC-25, TU-183, HSU-3, FADU, OEC-M1, SNU-1041 and SCC-15 cells	([104], 28093311)
DLEU1	miR-490-3p	SAS, Ca9–22, HSC-3, KON, MOT, HSC-4, OSC-19 and MON2 cells	([70], 30069008)
CASC2	miR-21	SCC-090 and SCC-25 cells	([105], 30467776)

**Fig. 5:**
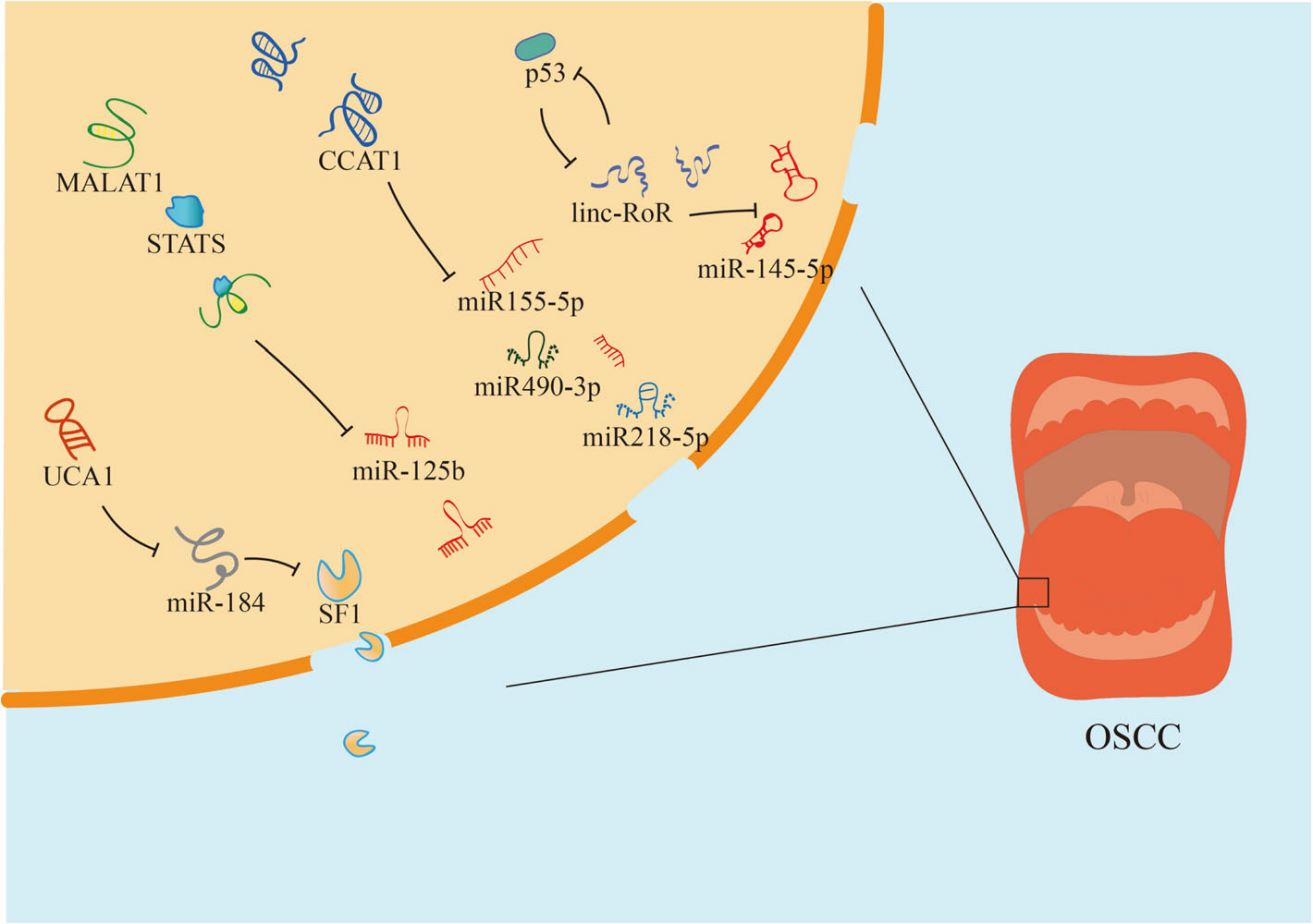
LncRNAs trigger OSCC through multiple regulatory signaling pathways. A variety of methods have been used to detect upregulation or downregulation of multiple lncRNAs in OSCC patients. These lncRNAs are affected by upstream regulatory factors or affect downstream factors to trigger carcinogenic or tumor suppressor signaling pathways

By using bioinformatic analysis, it had been confirmed that there were 160 differentially expressed lncRNAs between OSCC and normal controls. Moreover, lncRNA FTH1P3, PDIA3F and GTF2IRD2P1 affected the progression and metastasis of OSCC by triggering MMP1, MMP3, MMP9, PLAU and IL8 [163]. There were 21 lncRNAs were significantly related to overall survival (OS) and disease-free survival (DFS) [164]. Among these 21 lncRNAs, a significant positive correlation was observed between the signatures of 13 lncRNAs (TTC39A-AS1, RP11-93B14.9, AC012456.4, RP11-87C12.5, RP11-464F9.21, LINC01549, RP11-897 M7.1, AP003900.6, LINC01343, RP11-181E10.3, CTD-2545H1.2, RP11-796E2.4 and LINC01108) and OS/DFS, while the signatures of the remaining eight lncRNAs (AC007879.2, BOK-AS1, CTB-161 M19.4, CTD-2033A16.3, FAM95B1, RP11-1C8.7, RP11-285G1.14 and RP11-286E11.1) were significantly negatively correlated with OS and DFS [164]. The above signal path can be seen in Fig. 5.

## Potential clinical application of lncRNA in OSCC as biomarker

Numerous lncRNAs were aberrantly expressed in various cancers, and some lncRNAs seemed to have been more cancer-specific. Most of them were stable in body fluids and detectable in the plasma and urine of cancer patients. Their expression levels were indicative of the severity of cancers. All of these factors contributed to lncRNAs as noninvasive biomarkers and therapeutic targets for treatment of cancers [165]. LncRNAs were different from protein-coding genes in many respects. First, due to their greater abundance than protein-coding genes, a modulation in a larger number of lncRNA expression may be observed in each subtype of cancer, which provided a larger window for the detection of subtype-specific lncRNA-based biomarker. Second, subtype/tissue–specific lncRNA expressions were crucial for developing novel diagnostic biomarker and personalized therapy [166, 167]. Furthermore, given their participation in diverse cellular signaling pathways and tissue-specific expression, lncRNAs can be utilized to formulate novel strategies for specific cancer subtype diagnosis and targeting. The effects of several representative lncRNAs are shown in the Table 4.

**Table 4:** The main roles of the exemplified lncRNAs

LncRNA	Molecular functions	Mechanism
MALAT1	RNA splicing regulator	Sponges miR-125b and promotes STAT3 expression.
CCAT1	miRNA sponge	Sponges for miR-155-5p and let7b-5p.
MEG3	Transcriptional repressor	Suppresses Tumor via Wnt/β-catenin signaling pathway.
NEAT1	Chromatin modification	Downregulates miR-365 expression.
TUG1	miRNA sponge	Promotes OSCC via TUG1/miR-219/FMNL2 axis.
UCA1	Transcriptional activator	Promotes tumor invasion and metastasis possibly through Wnt/β-catenin signaling pathway.
Linc-RoR	miRNA sponge	Sponges for miR-145 to inhibit the expression levels of OCT4, NONOG and SOX2.

Some lncRNAs were already implicated as biomarkers, though some of them were in clinical trials (Table 5) [36, 103, 130, 137, 168]. For example, compared with the adjacent normal tissues, lncRNA C5orf66-AS1 expression was significantly decreased in OSCC tissues. LncRNA EGFR-AS1 was highly upregulated in the neck squamous cell carcinoma, and was speculated to be an OSCC biomarker [169]. Thus, lncRNAs appeared to be promising novel diagnostic and prognostic markers for a variety of cancers, however, there were still many challenges and validations required for their clinical applications. The utility of circulating and salivary lncRNAs as potential biomarkers gained interest in oral cancers. Plasma levels of HOTAIR and other two lncRNAs (lincRNA-p21 and GAS5) were measured by quantitative polymerase chain reaction, and it found that they were associated with the treatment response of 41 patients with head and neck cancer who underwent radical chemo radio therapy [170]. Maarabouni et al. [171] found higher expression of GAS5 in the patients with progressive disease when compared with those with the good clinical responses. Blood and saliva may provide novel insights into the establishment of new protocols for the detection patients with OSCC.

**Table 5:** LncRNA biomarker for different cancers

LncRNA	Ensembl ID	Sequence Name (*Homo sapiens*)	Biomarker in cancers	Reference (PMID)
HOTAIR	ENSG00000228630	hotair_hg_1	BrC, HCC, CoC, PaC, LuC, OC	([168], 26208723])
GAS5	ENSG00000234741	gas5_homosapiens_1	BrC, PrC, LuC, MPM	([36], 26634743])
MALAT1	ENSG00000251562	malat1_homosapiens_1	LuC, BlC, BrC, CeC, CoC, CoC, EnC	([114], 28837398])
CCAT1	ENSG00000247844	ccat1_hg_1	CoC, GasC, HCC, GalC, OC, BrC, LuC	([130], 27134049])
MEG3	ENSG00000214548	meg3_homosapiens_1	BlC, BMC, BrC, CeC, CoC, HCC, LuC, MC, PrC	([137], 22393162]
UCA1	ENSG00000214049	UCA1_hg_1	BlC, BrC, CoC, GasC, OC	([145], 26341664])
NEAT1	ENSG00000245532	neat1_homosapiens_1/2	LuC, EsC, LaC, CoC, HCC, PrC, BrC	([103], 28105699])

*BlC* Bladder cancer, *BMC* Bone marrow cancer, *BrC* Breast cancer, *CeC* Cervical cancer, *CoC* Colorectal cancer, *EnC* Endometrial cancer, *EsC* Esophageal cancer, *GalC* Gallbladder cancer, *GasC* Gastric cancer, *HCC* Hepatocellular cancer, *LaC* Laryngeal cancer, *LuC* Lung cancer, , *MPM* Malignant pleural mesothelioma, *MC* Meninges cancer, *OC* Ovarian cancer, *PaC* Pancreatic cancer, *PrC* Prostate cancer

Multiple lncRNAs were demonstrated to have tumorigenic effects via emerging technologies. LncRNAs, being large in size, may fold into complex secondary/tertiary structures and scaffolds, which may aid in cancer initiation and progression. Li et al. [172] disclosed the ceRNA network and indicated that two lncRNAs (PART1, TTTY14), four miRNAs (hsa-mir-133a, hsa-mir-135b, hsa-mir-196b, hsa-mir193b) and one transcription factor (MEIS1) might be closely related to OSCC tumorigenesis. After DNA damage in Tca8113 cell, HOTAIR mRNA expression increased, and further promoted Tca8113 cell proliferation. When HOTAIR mRNA expression was affected, Tca8113 cell proliferation was blocked in the G_2_/M or M phase [168]. This indicated that HOTAIR had an oncogenic role and might be an eligible target for OSCC treatment. OSCC patients were found to express higher levels of lncRNA-FOXCUT (a new lncRNA FOXC1 upstream transcript) and mRNA-FOXC1 (fork head box C1 gene) by RT-PCR detection. FOXCUT level was downregulated by siRNA, and FOXC1 level was also downregulated, indicating that FOXCUT was a regulator of FOXC1. Downregulation of FOXCUT and FOXC1 levels inhibited the expression of MMPs (preventing proliferation and migration of OSCC cell (Tca8113 and SCC-9)) and angiogenesis factor VEGF-A (blocking OSCC angiogenesis) [173, 174]. It also revealed that UCA1 had an oncogenic role in OSCC cells in vivo and in vitro [94].

Increased NEAT1 (nuclear paraspeckle assembly transcript 1) levels in OSCC tissues and cells were consistent with advanced TNM stage and poor survival of patients. High levels of NEAT1 antagonized miR-365 (a potential tumor suppressor or oncogene) expressions. Meanwhile, downregulation of NEAT1 levels inhibited cell proliferation and infiltration, suggesting that OSCC could be treated by modulating NEAT1/miR-365 levels [103, 175]. High expression levels of H19 in OSCC tissues were also found to be associated with TNM stage, nodal invasion and a shorter OS. Low expression of H19 can interfere with the proliferation of OSCC cell and inhibit tumor growth [176]. Yu et al. [177] demonstrated that the OS of low and high expression LINC00152 groups were 35 and 28 months, and the RFS were 29 and 26.5 months. There results indicated that LINC00152 may serve as an oncogene in OSCC, and might be a biomarker for early detection, treatment and prognosis prediction of OSCC. An expanded case-control study found that abnormal AC007271.3 levels were significantly associated with clinical stage of OSCC. It suggested that AC007271.3 could be novel circulating biomarkers for the determination of OSCC [178].

In addition to tumorigenic effects, some lncRNAs also played a role in inhibiting tumors. Yang et al. [179] found that GAS5 content in OSCC was lower than that in normal tissues, and suggested that the overexpression of GAS5 inhibited tumor proliferation, migration and invasion ability. Therefore, GAS5 may be clinically used as an anti-oncogene and provided a new target for the treatment of OSCC. Meanwhile, targeted knockout of HOTAIR can be used as a method of treating OSCC [100].

## LncRNAs in OSCC prognosis

Surgical resection was considered to be a promising treatment strategy for cancer patients at the early stages. However, recurrence after surgical resection was still a major cause of OSCC-related death. According to report, location, risk factors, clinical stage and treatment, etc. may affect the prognosis of OSCC patients [180]. Biomarkers that predicted the prognosis of patients early were urgently needed to be clinically demonstrated, but they were still not ideal.

Because of the unique role of lncRNAs in OSCC patients, the researchers turned their attention to these molecules. Dong et al. [105] found plasma levels of lncRNA CASC2 decreased in patients with local recurrence but increased in patients without recurrence. And lncRNA CASC2 overexpression promoted cancer cell proliferation. Therefore, CASC2 may participate in the prognosis of OSCC after surgical resection. FLJ22447, referred to LncRNA-CAF, was first found that upregulated CAF was associated with poor prognosis, suggesting it acts as a novel potential OSCC therapeutic target [106]. High expression of HNF1A-AS1 in OSCC samples suggested a poor prognosis, while HNF1A-AS1 deletion inhibited the proliferation, migration and EMT of OSCC cells [97]. Zhou et al. [88] found high MALAT1 levels in 54 OSCC tumor samples and individuals accompanied the poor prognosis. LINC01133 was downregulated in OSCC; higher expression of LINC01133 in OSCC was correlated with less metastasis and better prognosis [181].

The undifferentiated OSCC exhibited a high linc-RoR expression. The phenomenon can be attributed to linc-RoR overexpression interacting with miR-145, causing increased pluripotent transcription factors that regulated the cellular differentiation. High levels of linc-RoR were detected in tissue samples from tumor relapse and drug-resistant patients, suggesting that clinical detection of linc-RoR level predicted the prognosis and therapeutic effects of OSCC [101]. Furthermore, result of the data in the TCGA database concluded that CCAT1 overexpression was associated with poor survival, suggesting that high levels of CCAT1 presented poor therapeutic responses [90]. The expression levels of SOX21-AS1 in OSCC cells were significantly reduced when compared with adjacent normal tissues. In addition, the data showed that the low expression level of SOX21-AS1 was associated with an advanced stage (*P* = 0.047), large tumor size (*P* = 0.033), and poor survival in OSCC patients (*P* = 0.002). These results suggested that low levels of SOX21-AS1 expression may indicate the poor prognosis in OSCC patients [182]. Multivariate Cox proportional hazards regression analyses were used to further determine AC012456.4 low expression as an independent prognostic risk factor (DFS: *P* = 0.004, HR = 0.600, 95% CI = 0.423–0.851; OS: *P* = 0.002, HR = 0.672, 95% CI = 0.523–0.863). Moreover, AC012456.4 was pointed out for the first time that it can be used as a novel molecular target for clinical diagnosis, treatment and prognosis for OSCC patients [164].

These lncRNAs work together to form a complex regulatory network, and the credibility of the prognosis is predictive. It is urgent to explore one or a group of lncRNAs as a prognostic indicator in the future.

## Future expectations

Considering that lncRNAs have high cell-type specificity, they have been utilized for selectively kill tumor cells without damaging normal cells. H19-DTA (BC-819), a DNA plasmid that carries the gene for diphtheria toxin-A, is used to target H19 overexpressing cancer cells under the regulation of the H19 promoter sequence. The injection of H19-DTA reduces the size of multiple tumor types by inducing the expression of diphtheria toxin [183]. Lavie et al. [184] conducted a phase 1-2A multi-centric trial included 14 eligible ovarian/peritoneal cancer patients. H19-DTA was injected into patients by intra-peritoneal instillations for a maximum of 6–9 weeks. During the study, no dose-limiting toxicities were observed and median survivals of 3.2, 5.3 and 6.5 months were observed for the 60, 120 and 240 mg cohorts, respectively. These results indicated that H19-DTA given locally can provide ancillary therapeutic effects for systemic chemotherapy in ovarian or peritoneal cancer. Gofrit rt. al. [185] included 47 patients with recurrent, multiple nonmuscle invasive bladder tumors in a phase 2b trial. Patients expressing H19 received a 6-week induction course of intravesical H19-DTA. 33% of patients showed complete tumor ablation and 64% were no new tumors at 3 months. At the same time, the median time to recurrence was also significantly prolonged in responding patients. It was concluded that H19-DTA served as a potential medication for bladder cancer. These results are gratifying. Because it allows us to speculate that other lncRNAs have similar therapeutic effects, although they have not been discovered at this stage.

LncRNA research in OSCC was also still incipient. The upregulated and downregulated lncRNA profiles in oral cancers should be established [186], but the differentially expressed lncRNAs needed to be functionally evaluated in the context of the cells investigated. Meanwhile, the road to exploration was not going well. For example, many lncRNAs are located in the nucleus and are difficult to knock out [187].

Recently, the successful application of CRISPR-Cas9 (clustered regulatory interspaced short palindromic repeats/CRISPR-associated protein 9) technology to model plants has given us new inspiration. CRISPR-Cas9 is considered to be a bacterial defense mechanism against phage infection and plasmid transfer and simple, versatile and promising genome editing technique [188]. Use of CRISPR-Cas9 for the treatment of schizophrenia has once again enhanced our confidence in treating cancer [189]. The lncRNAs editing protocol based on CRISPR-Cas9 technology has been widely used in a variety of diseases, including cancer, albeit there are currently limitations in this system [190]. There is not enough research to prove that Cas9 technology can be applied to OSCC, but some theoretical connections let us see hope [191]. This technology is expected to use to treat OSCC and clarify the relevant mechanisms.

Exosomes, nanosized (30-100 nm) membrane microvesicles, can act as messengers in the interstitial to establish communication between cancer cells and basal cells [192]. There is evidence that exosomes regulate tumor growth and metastasis through inclusions, containing lncRNAs (Table 6) [193–203], and serve as noninvasive biomarkers for early detection, diagnosis, and treatment of cancer patients [204]. Zhang et al. [193] found that the MALAT-1 content in exosomes of lung cancer patients was overexpressed. Functionally, serum exosome-derived MALAT-1 promoted tumor growth and metastasis. It indicated that MALAT-1 in exosomes can be used as a noninvasive biomarker for diagnosis and prognosis of non-small cell lung cancer [193]. Therefore, finding out the specific lncRNAs in the exosomes of patients with OSCC is the direction of future exploration.

**Table 6:** Exosome lncRNAs in different cancers

LncRNA	Cancer type	Function in cancer cells	Reference (PMID)
MALAT1	LuC	Proliferation, migration	([193], 28623135)
UCA1	BlC; CoC	Proliferation, migration, invasion	([194], 28841829); ([195], 29948578)
ZFAS1	GasC;	Proliferation, migration	([196], 28285404)
CRNDE-h	CoC	Metastasis	([197], 27888803)
HOTAIR	BlC	Migration, invasion	([198], 26800519)
91H	CoC	Migration, invasion	([199], 29410604)
H19	HCC	Angiogenesis	([200], 26272696)
CCAT	Glioma	Angiogenesis	([201], 28656228)
LINC-ROR	HCC	Tumor resistance regulator	([202], 24918061)
ARSR	RC	Sunitinib resistance	([203], 27117758)

*BlC* Bladder cancer, *CoC* Colorectal cancer, *GasC* Gastric cancer, *HCC* Hepatocellular cancer, *LuC* Lung cancer, *RC* Renal cancer

## Conclusion

It is imperative to crack the carcinogenic or tumor suppressor mechanism of lncRNAs, which is of great significance for the diagnosis and treatment of cancer by utilizing lncRNAs. A curated collection and summary of deregulated lncRNAs in cancer is essential to thoroughly understand the mechanisms and functions of lncRNAs. The low accessible amount of lncRNAs poses an obstacle to detection. At the same time, the mechanism of interaction between lncRNAs is poorly understood. Problems such as these are believed to be solved using animal models and new generation of technologies. LncRNA studies in oral cancer are expected to undergo a vast expansion in decades. Furthermore, we suggest strategies to accelerate the pace from the bench to the bedside.

## Abbreviations

3-UTRs: 3-untranslated regions
ATG: Autophagy-related gene
CCAT1: Colon Cancer Associated Transcript 1
CDKN2A: Cyclin-dependent kinase inhibitor 2a
ceRNA: competing endogenous RNA
circRNAs: circular RNAs
CRISPR-Cas9: Clustered regulatory interspaced short palindromic repeats/CRISPR-associated protein 9
DFS: Disease-free survival
DLEU1: Deleted in lymphocytic leukemia 1
DPD: Dihydropyrimidine dehydrogenase
EGFR: Epidermal growth factor receptor
EMT: Epithelial-mesenchymal transition
FOXC1: Fork head box C1 gene
FTH1P3: Ferritin heavy chain 1 pseudogene 3
Gtl2: Gene trap locus2
HIOECs: Human immortalized oral epithelial cells
HOTAIR: HOX transcription antisense RNA
HPV: Human papillomavirus
lncRNAs: long non-coding RNAs
MALAT1: Metastasis-associated lung adenocarcinoma transcript 1
MAP 1 LC3B: Microtubule-associated protein 1 light chain 3B
MEG3: Maternally expressed gene 3
miRNAs: microRNAs
ncRNAs: non-coding RNAs
NOTCH1: Notch homolog 1 genes are translocation-associated
OS: Overall survival
OSCC: Oral squamous cell carcinoma
*P. gingivalis*: *Porphyromonas gingivalis*
STAT3: Signal transducer and activator of transcription 3
UCA1: Urothelial cancer associated 1
